# Ultra-Short DNA Fragments Undergo A-to-B Conformational Transitions Revealed by FTIR Spectroscopy

**DOI:** 10.3390/ijms27041876

**Published:** 2026-02-15

**Authors:** Kristina Serec, Josip Basić, Martin Bobek, Antonia Lovrenčić, Lucija Totić, Sanja Dolanski Babić

**Affiliations:** Department of Physics and Biophysics, School of Medicine, University of Zagreb, 10000 Zagreb, Croatia

**Keywords:** ultra-short DNA, DNA conformation, magnesium ions, ionic environment, electrostatic screening, FTIR spectroscopy, vibrational spectroscopy

## Abstract

Understanding interactions between cations and DNA is essential for elucidating the structural dynamics of this fundamental biomolecule. While B-DNA is well known to dominate in long genomic DNA under physiological ionic conditions, its stability in very short DNA fragments—particularly in dilute solutions and in crude oligonucleotide preparations—has remained largely unexplored. Previous spectroscopic studies have primarily focused on long DNA, highly purified oligonucleotides, or high-salt environments, where collective polyion effects dominate. In contrast, the present results demonstrate that even in the absence of chain overlap and under low-salt conditions, Mg^2+^ ions efficiently stabilize the B-form by screening phosphate–phosphate electrostatic repulsion at the intrachain level. The ability to induce an A-to-B transition in crude, ultra-short DNA fragments highlights the fundamental role of divalent counterions in governing DNA conformation and establishes a lower bound for the length scale at which B-DNA can be stabilized. These findings are particularly relevant for dilute biological systems, fragmented DNA samples, and analytical protocols where short DNA fragments and low ionic strength are unavoidable.

## 1. Introduction

Deoxyribonucleic acid (DNA) is a highly charged biopolymer whose structure and biological function are governed by interactions with its surrounding ionic environment. These interactions are essential for fundamental biological processes such as DNA replication, repair, and transcription [1].

Electrostatic interactions mediated by cations play a central role in determining DNA stability and conformation. Within the framework of counterion condensation theory, multivalent cations such as Mg^2+^ accumulate close to the negatively charged phosphate backbone, effectively screening electrostatic repulsion and stabilizing the DNA double helix [2]. In addition to electrostatic screening conditions, both DNA length and DNA concentration in solution critically influence the strength and nature of these electrostatic interactions.

DNA length is particularly important for its electrostatic properties. Long DNA chains can engage in extensive self-interactions, leading to aggregation and condensation phenomena that complicate the analysis of ion-mediated effects [3]. In contrast, short DNA fragments exhibit limited polyion self-interaction, providing a simpler model system for studying how cations such as Mg^2+^ or Mn^2+^ influence DNA structure without the confounding effects present in long-chain systems. Previous studies have demonstrated that polyion length significantly affects electrostatic screening and the overall stability of DNA structures [4].

The behavior of charged biopolymers such as DNA in solution also strongly depends on polymer concentration. Polyelectrolyte theory predicts distinct conformational and thermodynamic regimes in dilute and semidilute solutions, governed by both DNA concentration and fragment length [5]. Consequently, the structural properties of double-stranded DNA are determined by an interplay between intrinsic polyion characteristics and extrinsic buffer properties, including the type and concentration of surrounding ions.

In the literature, DNA structural studies are often performed using a wide range of DNA lengths, concentrations, buffer compositions, and experimental techniques. Spectroscopic methods such as Fourier-transform infrared (FTIR) spectroscopy, circular dichroism, UV–visible spectroscopy, and dielectric spectroscopy each offer distinct advantages but also impose specific constraints on accessible concentration ranges and ionic conditions [6,7,8]. As a result, meaningful comparisons across studies remain challenging, and it is often difficult to disentangle intrinsic polyion effects from buffer- or ion-induced behavior. This issue becomes particularly pronounced at low DNA concentrations relevant to biological samples and DNA extraction protocols, where variations in ionic environment can substantially influence DNA behavior.

In addition to these methodological challenges, understanding the limits of DNA conformational stability as a function of fragment length is of growing importance for a broad range of applications [9,10,11,12,13,14]. Biologically relevant ultra-short DNA fragments, typically tens of base pairs in length, are commonly encountered in fragmented and degraded DNA samples obtained following standard extraction and fragmentation protocols, including cell-free DNA, as well as during apoptotic and necrotic processes [1]. In addition to their biological relevance, short and ultra-short DNA sequences are widely employed as sequence-defined synthetic oligonucleotides in biosensing and analytical applications, as well as in DNA self-assembly and nanostructure design [11]. Despite their importance in both biological contexts and experimental applications, the electrostatic properties of ultra-short DNA fragments, particularly those governing intrachain screening, conformational stability, and A–B transitions, remain insufficiently explored. Extensive spectroscopic studies of DNA conformation have predominantly focused on long genomic DNA, semidilute regimes, and/or high-salt conditions [9,12,13,15,16,17]. In many cases, DNA fragment length is either not explicitly specified or spans a broad and uncontrolled range, potentially obscuring length-dependent electrostatic and conformational effects. This study directly addresses this gap. A systematic understanding of how DNA length, concentration, and ionic environment jointly determine DNA conformation is therefore essential for interpreting experimental results and for the rational design of DNA-based systems in both fundamental and applied research.

In our previous work, we systematically investigated the influence of added salts on the structure and conformation of long double-stranded DNA using FTIR spectroscopy applied to DNA thin films [15,16]. These studies demonstrated that divalent magnesium ions predominantly interact electrostatically with the negatively charged phosphate backbone, leading to effective charge screening and stabilization of the B-form of DNA over a broad range of Mg^2+^ concentrations. At sufficiently high magnesium content, signatures of more compact DNA conformations were observed, reflecting enhanced electrostatic screening and local charge neutralization [15]. In addition to salt-induced effects, we examined the role of hydration and drying dynamics on the conformational state of DNA thin films. By systematically controlling the drying time and hydration level, we showed that changes in water content strongly affect vibrational signatures associated with A- and B-form DNA, while certain phosphate vibrations primarily reflect hydration rather than conformational transitions. These results established FTIR spectroscopy of DNA thin films as a sensitive and reliable tool for disentangling hydration-driven and ion-mediated structural effects in long-chain DNA systems [16]. Complementary to these structural studies, we previously investigated short-fragment DNA in dilute aqueous solutions using dielectric spectroscopy. These experiments revealed pronounced differences between the dielectric response of short and long DNA, highlighting the critical role of DNA length and concentration in governing electrostatic screening and counterion dynamics. The observed dielectric relaxation modes were successfully interpreted within the framework of polyelectrolyte theory, demonstrating that short DNA fragments exhibit distinct fundamental length scales and screening behavior compared to long, genomic DNA [17].

In this study, we focus explicitly on ultra-short DNA fragments and systematically investigate how variations in DNA concentration and the concentration of added magnesium ions influence their vibrational properties and conformational state using FTIR spectroscopy. Ultra-short DNA refers to fragments comprising several helical turns, corresponding to a fragment length range of 15–50 base pairs. In such a regime, DNA fragments are sufficiently short to suppress long-range flexibility while remaining long enough to sustain a well-defined double-helical structure. Given that one helical turn of B-DNA comprises approximately 10–10.5 base pairs, the investigated fragments span multiple helical turns and thus permit meaningful investigation of A–B conformational transitions. In addition, such ultra-short DNA fragments are commercially available (crude oligonucleotides, i.e., lacking sequence-specific purification or chemical stabilization), chemically well-defined, and reproducible, enabling systematic preparation of multiple samples under controlled conditions. By spanning DNA concentrations from 0.1 to 5 g/L, the present study systematically explores the deeply dilute regime relevant to fragmented DNA while approaching the overlap threshold at higher concentrations. This allows the influence of polyion concentration on intrachain electrostatic stabilization to be directly assessed. By independently tuning both polyion concentration and ionic environment within the dilute regime, the present approach isolates intrachain electrostatic screening effects that are not accessible in long-DNA or semidilute systems. In this way, the study bridges our previous thin-film FTIR investigations and dielectric spectroscopy studies, providing new insight into ion-mediated conformational behavior and A–B transitions in ultra-short DNA fragments under conditions directly relevant to fragmented biological DNA and dilute experimental environments.

## 2. Results

To investigate how variations in DNA concentration and divalent cation concentration affect the vibrational signatures of short-chain DNA, a series of aqueous DNA solutions containing magnesium cations was prepared. In total, fifteen solutions were analyzed, combining three DNA concentrations (0.1 g/L, 1 g/L, and 5 g/L) with five different cation/phosphate molar concentration ratios, defined as r = [Mg]/[P]: r = 0.1, 0.5, 1, 1.5, and 2. To probe how progressive addition of Mg^2+^ cations affect the infrared vibrational properties of short-chain DNA, the results are organized as follows. In Section 2.1, we examine the effect of DNA concentration under conditions of weak screening (r = 0.1), moderate screening (r = 1), and strong screening (r = 2). In Section 2.2, we focus on the effect of increasing cation content at a fixed DNA concentration.

Extensive assignment of vibrational bands of long-fragment DNA, together with a systematic comparison of our results with previously published FTIR studies, has been presented in our previous work [16]. For convenience, the most relevant band assignments in the sugar–phosphate region used in the present analysis are summarized in Table 1. The bands at approximately 1232, 1185, 893, 860, 837, and 805 cm^−1^ are commonly regarded as conformation-related markers in FTIR spectra of DNA. A detailed discussion of the origin, sensitivity, and limitations of these bands lies beyond the scope of the present work; therefore, the reader is referred to our previous publication [15,16] for a comprehensive analysis, as well as the references cited therein.

### 2.1. Effects of DNA Concentration

**Figure 1:**
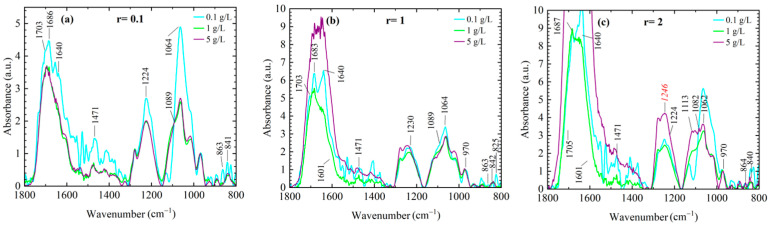
Average FTIR spectra of ultra-short DNA thin films obtained by drying DNA solutions in the presence of Mg^2+^ ions, recorded in the 1800–800 cm^−1^ spectral range, illustrating the effect of DNA concentration under different electrostatic screening conditions. Spectra are shown for three DNA concentrations: 0.1 g/L (light blue), 1 g/L (green), and 5 g/L (purple), at magnesium-to-phosphate ratios r = [Mg]/[P] of (**a**) 0.1, (**b**) 1, and (**c**) 2. Numbers above the spectra indicate key vibrational bands, including asymmetric and symmetric PO_2_^−^ stretching modes (~1224 and ~1089 cm^−1^). Conformation-sensitive bands associated with A- and B-form DNA are located in the 900–800 cm^−1^ region (A-form: ~863 cm^−1^; B-form: ~840 cm^−1^).

Figure 1 illustrates the intricate interplay between DNA concentration and ionic environment. In particular, Figure 1a (r = 0.1), corresponding to weak electrostatic screening by Mg^2+^ ions, reveals that DNA concentration has a significant impact on the structure of short-chain DNA. In the low-screening limit (r = 0.1) and at low DNA concentration (0.1 g/L), short-chain DNA is less stable compared to samples with moderate and high DNA concentrations (1 g/L and 5 g/L, respectively). Increased absorption of base vibrational bands and phosphate-related modes, namely the asymmetric and symmetric phosphate stretching bands at 1224 cm^−1^ and 1089 cm^−1^, respectively, as well as the C–O phosphodiester band at 1062 cm^−1^, indicates reduced structural stability of short-chain DNA under these specific conditions of low DNA concentration and low salt content [18,19,20]. In particular, the spectra display broadened vibrational bands and increased variability between measurements, most notably in the base vibration region and the conformation-related region (Figure 1).

The spectral range between 900 and 800 cm^−1^, which contains conformation-sensitive bands associated with deoxyribose vibrations, exhibits the appearance of multiple overlapping features, suggesting a mixed or dynamically fluctuating population of geometries. The strongest bands in this region correspond to vibrations commonly associated with A-form DNA (863, 820, and 805 cm^−1^), indicating partial A-form character under these conditions; however, the presence of additional overlapping features indicates that other geometries cannot be excluded. These spectral characteristics reflect enhanced sensitivity of ultra-short DNA to weak electrostatic screening and ensemble averaging over a less stable conformational population.

Importantly, this regime of low DNA concentration and low salt content provides a well-defined reference state for investigating the effects of increased polyion concentration and divalent counterion screening on conformational stability and A–B transitions in ultra-short DNA.

With increasing DNA concentration, polyions increasingly engage in self-interactions and polyion–polyion interactions, leading to enhanced overall structural stability and a conformational transition toward the B-form of DNA. Notably, the spectra obtained at DNA concentrations of 1 g/L and 5 g/L are essentially identical at r = 0.1, indicating that further increases in DNA concentration do not induce additional conformational changes under weak screening conditions.

Figure 1b (r = 1), corresponding to moderate electrostatic screening, shows that changes in DNA concentration have a reduced effect on phosphate vibrations and overall DNA conformation. By increasing the magnesium-to-phosphate concentration ratio from 0.1 to 1, the ionic environment of DNA molecules becomes such that, already at a DNA concentration of 0.1 g/L, spectral changes relative to Figure 1a indicate stabilization of the B-form of DNA.

Figure 1c (r = 2) reveals that conformational changes associated with DNA concentration become pronounced again under strong screening conditions and high DNA content. The combined presence of a high concentration of Mg^2+^ cations and a high density of phosphate groups enhances self-interactions and polyion–polyion interactions. This is evidenced by significant changes in both asymmetric and symmetric phosphate stretching vibrations, as well as pronounced variations in conformation-sensitive bands near 864 cm^−1^ and 836 cm^−1^.

### 2.2. Effects of the Magnesium-to-Phosphate Ratio

**Figure 2:**
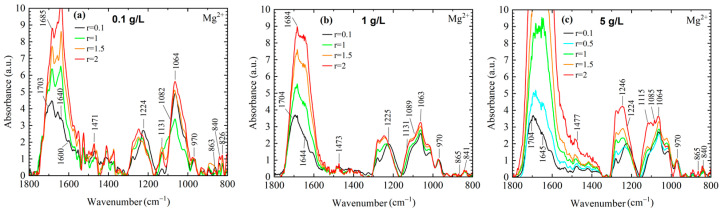
Average FTIR spectra of ultra-short DNA thin films obtained by drying DNA solutions in the presence of Mg^2+^ ions, recorded in the 1800–800 cm^−1^ spectral range for different molar concentration ratio r = [Mg]/[P]: 0.1 (black), 1 (green), 1.5 (orange) and 2 (red). The DNA concentration is represented by: (**a**) 0.1 g/L, (**b**) 1 g/L and (**c**) 5 g/L. Numbers above the spectra denote key vibrational bands of the r = 0.1 (black), corresponding to the lowest salt content including the asymmetric and symmetric PO_2_^−^ stretching modes at ~1224 cm^−1^ and ~1089 cm^−1^, respectively. Bands associated with A- and B-form DNA are located in the 900–800 cm^−1^ region (A-form: ~863 cm^−1^; B-form: ~840 cm^−1^).

In Figure 2a, the spectral changes reveal a multitude of A-conformation–related bands. At r = 0.1, increased absorption of the symmetric and asymmetric phosphate stretching bands indicates that ultra-short DNA fragments adopt a destabilized double-stranded conformation, predominantly associated with the A-form of DNA. In addition, changes observed in the base vibration bands, as well as in the band at 1131 cm^−1^, point to modifications in hydrogen-bonding interactions.

For DNA solutions with a concentration of 1 g/L, the spectra do not exhibit pronounced changes upon increasing the ratio cation magnesium (Figure 2b). DNA remains in a stable B-form even at the lowest ratio, as indicated by the positions and intensities of the A- and B-form marker bands. In contrast, DNA solutions with a concentration of 5 g/L show pronounced changes in both the symmetric and asymmetric phosphate bands, as well as in base-associated vibrations, with particularly dramatic effects observed at r = 2. These spectral changes are further analyzed in detail in the Discussion section in order to avoid repetition and to enhance overall readability.

## 3. Discussion

Understanding how counterions stabilize DNA structure is essential for elucidating the behavior of short DNA fragments in biological and physicochemical environments. Namely, ultra-short DNA fragments, comprising several helical turns, closely mimic biologically relevant DNA encountered in degraded samples, cell-free DNA, and common DNA extraction protocols. Beyond biological contexts, short and ultra-short DNA sequences are also widely employed as synthetic oligonucleotides in biosensing and analytical applications, as well as in DNA self-assembly and nanostructure design [11].

Ultra-short DNA fragments exhibit reduced thermodynamic stability compared to long DNA due to the loss of cooperative base stacking and end effects, which become increasingly important as fragment length decreases [2]. Thus, their conformational behavior is generally assumed to be less stable and less controllable, particularly under conditions of low ionic strength and weak electrostatic screening [4,6,17]. Consequently, the observation of a well-defined A-to-B conformational transition and a stable B-form in such systems is non-trivial and underscores the robustness of counterion-mediated stabilization mechanisms. Our results demonstrate that dilution destabilizes the B-form, while increasing Mg^2+^ concentration counteracts this effect through enhanced electrostatic screening of the phosphate backbone. These findings establish FTIR spectroscopy as a sensitive probe of ion-induced conformational transitions in ultra-short DNA, providing new insight into the fundamental limits of B-DNA stability in short, dilute, and biologically relevant DNA systems.

Remarkably, we show that the addition of Mg^2+^ counterions stabilizes a well-defined and spectroscopically stable B-form even in DNA fragments as short as 15–50 bp and under dilute solution conditions. This stabilization occurs despite the use of ultra-short oligonucleotides and relatively low salt content, highlighting the efficiency of divalent counterions in promoting the A-to-B conformational transition.

Comparison of ultra-short DNA at r = 0.1 with long DNA at r = 0.15 shows that similar low-screening ionic conditions do not lead to the same structural response [15]. While in long DNA even a small Mg^2+^ content efficiently stabilizes the B-form through effective electrostatic screening of the phosphate backbone, ultra-short DNA fragments exhibit a markedly weaker stabilization under comparable conditions. This difference reflects the limited ability of short polyions to sustain long-range electrostatic correlations and collective conformational response [15].

These observations suggest that, in addition to the strength of electrostatic screening, the concentration regime of the DNA solution plays a crucial role in determining the balance between electrostatic screening and conformational stability, motivating a closer examination of dilute and semidilute regimes. A dilute solution is defined as a solution with a low concentration of polyelectrolytes in which individual chains do not overlap because they are separated by sufficiently large distances from one another [5]. In such dilute solutions, intrachain interactions dominate over interchain interactions. As the polyelectrolyte concentration increases, the chains begin to overlap, enabling the emergence of new fundamental spatial length scales.

The characteristic concentration at which polyelectrolyte chains begin to overlap is referred to as the overlap concentration, c*. Solutions with concentrations c > c*, in which polymer chains overlap, are termed semidilute solutions, whereas solutions with concentrations c < c* are classified as dilute solutions. The concept of the overlap concentration was first introduced by de Gennes and later quantitatively refined by Dobrynin and co-workers using a different theoretical approach [3,5].

In a simplified picture, a DNA molecule can be regarded as a chain confined within a spherical volume whose diameter corresponds to the contour length of the chain, L_c_. The overlap concentration c* is then defined as the DNA concentration at which these volumes begin to touch. Consequently, solutions with c > c* correspond to the semidilute regime, while those with c < c* remain in the dilute regime.

**Figure 3:**
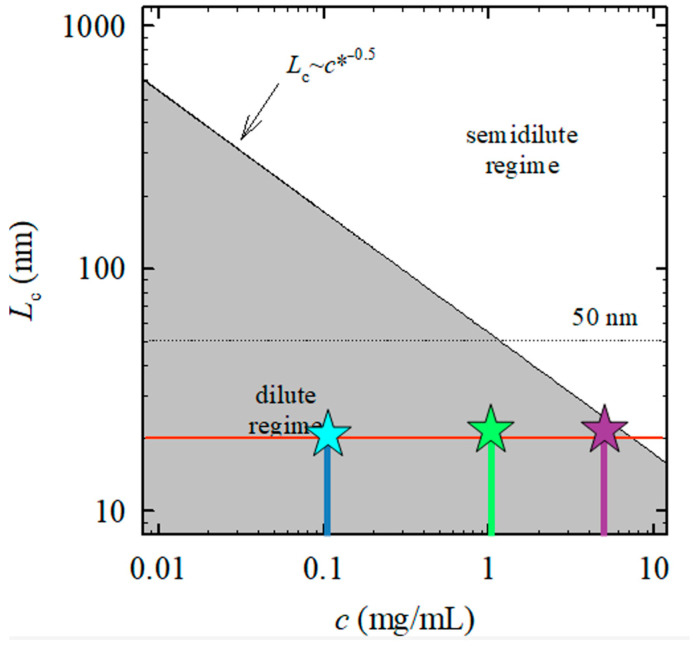
The solid gray line denotes the boundary between dilute and semidilute regimes, corresponding to the overlap concentration c*, defined by the DNA contour length and the DNA concentration in solution. Asterisks denote the maximum overlap concentrations for ultra-short DNA solutions at 0.1 g/L (light blue), 1 g/L (green), and 5 g/L (purple).

The diagram shown in Figure 3 demonstrates that all fifteen ultra-short DNA solutions investigated in this study fall within the dilute regime, with the lowest concentration of 0.1 g/L being particularly deep in this regime. The contour length of ultra-short DNA fragments considered here ranges from approximately 5 to 17 nm, whereas long DNA molecules typically exhibit contour lengths between 700 and 7000 nm. This pronounced difference in contour length results in markedly different overlap concentrations and underlies the fundamentally distinct electrostatic and conformational behavior of ultra-short and long DNA systems. Within the dilute regime (c < c*), where ultra-short DNA fragments do not overlap, the FTIR spectra primarily reflect intrachain properties and local electrostatic interactions between the phosphate backbone and surrounding counterions. In this regime, conformational changes induced by magnesium ions are manifested mainly through variations in phosphate stretching vibrations and A/B-form marker bands, indicating changes in local backbone geometry rather than collective structural rearrangements. The pronounced sensitivity of the spectra to DNA concentration at low Mg^2+^ content (e.g., r = 0.1) thus arises from the limited electrostatic screening and the absence of significant interchain interactions.

To conclude, within the investigated length range (15–50 bp, corresponding to contour lengths of approximately 5–17 nm), the observed behavior for low DNA concentrations is governed primarily by the absence of chain overlap and the predominance of intrachain electrostatic interactions. Accordingly, slightly longer DNA fragments are expected to exhibit qualitatively similar ion-mediated stabilization trends under comparable ionic conditions, provided that the system remains within the corresponding region of the dilute regime (c < c*).

In contrast, although the ultra-short DNA solutions investigated here remain formally within the dilute regime, increasing DNA concentration drives the system closer to the overlap threshold. This proximity enhances effective electrostatic coupling between fragments, which is reflected in the FTIR spectra by reduced differences between higher DNA concentrations (1 g/L and 5 g/L) and by a stabilization of B-form marker bands even under weak screening conditions. In this sense, increasing DNA concentration partially mimics the effect of increased ionic screening, consistent with the observed convergence of spectra at higher concentrations.

For long DNA molecules, whose contour lengths are orders of magnitude larger, the overlap concentration is significantly lower, and semidilute conditions are readily achieved. Under such conditions, FTIR spectra are dominated by collective polyion–polyion interactions, leading to a coherent stabilization of the B-form even at relatively low Mg^2+^ content. The comparison between ultra-short and long DNA therefore highlights that the same FTIR spectral markers may originate from fundamentally different physical mechanisms, depending on whether intrachain or interchain electrostatic interactions dominate. As discussed by Cherstvy [4], counterion-mediated electrostatic screening in DNA systems is governed not only by ionic strength but also by polymer length and spatial confinement, resulting in fundamentally different stabilization mechanisms for short and long DNA fragments.

At the atomic level, the observed A-to-B conformational transition is consistent with established models in which divalent counterions such as Mg^2+^ preferentially associate with the phosphate backbone, thereby reducing phosphate–phosphate electrostatic repulsion and stabilizing backbone geometries characteristic of the B-form. Molecular dynamics simulations of ion–DNA interactions and A-to-B conformational transitions support this mechanism [21]. However, it is worth noting that constructing a simulation framework capable of simultaneously reproducing the full range of experimental parameters explored here, including ultra-short DNA fragments, dilute concentration regimes, variable polyion overlap, and controlled ionic environments, while also providing direct correspondence to spectroscopic signatures, remains highly challenging.

**Figure 4:**
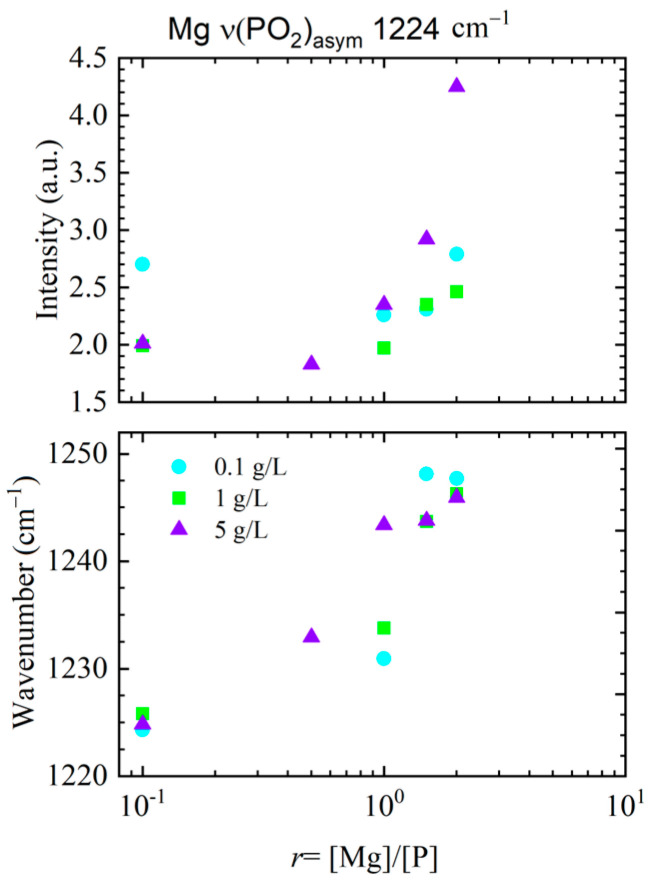
Dependence of the asymmetric phosphate stretching vibration (~1224 cm^−1^) on the magnesium-to-phosphate molar concentration ratio r = [Mg]/[P] for ultra-short DNA fragments. Upper panel: variation in band intensity. Lower panel: corresponding shift in the band position. Data are shown for DNA concentrations of 0.1 g/L (circles), 1 g/L (squares), and 5 g/L (triangles).

The trends discussed above are further supported by a quantitative analysis of the asymmetric phosphate stretching vibration, PO_2_^−^, shown in Figure 4. Both the intensity and the peak position of this band exhibit a systematic dependence on the magnesium-to-phosphate ratio r, reflecting changes in electrostatic screening and local backbone environment. For all DNA concentrations, increasing r leads to an increase in band intensity accompanied by a shift in the peak position toward higher wavenumbers, consistent with enhanced charge screening and reduced electrostatic repulsion between neighboring phosphate groups.

Notably, the magnitude of these changes depends on DNA concentration. At low DNA concentration (0.1 g/L), the response is relatively modest, indicating that screening effects primarily modify local intrachain interactions. In contrast, at higher DNA concentrations (1 g/L and 5 g/L), the changes become more pronounced, particularly at larger r, in agreement with the emergence of collective electrostatic effects discussed above. These results corroborate the conclusion that, even for ultra-short DNA fragments, phosphate vibrations provide a sensitive probe of the balance between polyion concentration and ionic screening.

The observed shift in the asymmetric phosphate stretching vibration with increasing Mg^2+^/phosphate ratio is evidence of pronounced magnesium interaction with the backbone phosphates and is primarily attributed to electrostatic screening of the negatively charged DNA backbone by divalent counterions, accompanied by reorganization of the local hydration environment. In our previous FTIR studies on long DNA and highly hydrated DNA thin films, we demonstrated that Mg^2+^-induced spectral changes are fully reversible upon ion depletion, with original vibrational signatures being restored [20]. This reversibility strongly supports a non-specific, water-mediated electrostatic interaction mechanism rather than direct, site-specific Mg^2+^ coordination. Within this framework, changes in phosphate band position and intensity reflect modifications of the local electrostatic and hydration landscape surrounding the backbone, rather than the formation of stable Mg^2+^–phosphate complexes.

Overall, dilution of DNA solutions reduces the polyion concentration and thus weakens the stability of the B-form of DNA, affecting the spatial organization of phosphate groups as well as the preservation of base pairing and hydrogen-bond integrity. Our results demonstrate that increasing the magnesium salt content counteracts this effect by enhancing electrostatic screening, thereby promoting the structural and conformational stability of ultra-short DNA fragments even at low DNA concentrations. However, at high DNA concentration and under conditions of strong screening, although the system formally remains within the dilute regime, pronounced spectral changes are observed at r = 2, affecting all major vibrational bands. This indicates that, under these conditions, collective electrostatic effects and enhanced polyion–polyion interactions become significant, highlighting the subtle balance between DNA concentration, ionic screening, and conformational stability in ultra-short DNA systems.

The present findings are particularly relevant for understanding biological and applied systems involving fragmented and ultra-short DNA, such as DNA obtained following standard extraction and fragmentation protocols or released during apoptotic and necrotic processes, as well as to DNA samples used in analytical and diagnostic assays. In DNA–nanoparticle assemblies and biosensor platforms, short DNA fragments are often employed at low concentrations and under non-physiological ionic conditions, where conformational stability cannot be assumed a priori.

The identification of a well-defined low-concentration, weak-screening regime as a reference state, together with the demonstrated stabilization induced by Mg^2+^ ions, provides a useful framework for the rational design of experiments involving short DNA in dilute environments. From a broader perspective, the results presented in this work demonstrate that careful control of both DNA concentration and the ionic environment is essential for achieving reproducible and well-defined DNA structural states. While the present work focuses on random-sequence DNA and Mg^2+^ as a representative divalent counterion, this experimental framework may be extended to sequence-defined oligonucleotides, other ions, and broader concentration regimes to further explore the interplay between polyion length, ionic screening, and conformational stability, as well as potential sequence- and ion-specific contributions to electrostatic stabilization in ultra-short DNA systems.

## 4. Materials and Methods

### 4.1. Preparation of DNA Solutions and Thin Films

Deoxyribonucleic acid (DNA) from herring sperm in powder form (double-stranded DNA), with an estimated fragment length of 5–17 nm (corresponding to short DNA fragments of 15–50 base pairs), was obtained from Sigma-Aldrich Corp. (St. Louis, MO, USA). Ultrapure Milli-Q water with a declared conductivity of 0.056 μS/cm was used in all experiments. Magnesium chloride hexahydrate (MgCl_2_·6H_2_O), obtained from Sigma-Aldrich Corp. (St. Louis, MO, USA), was used as the source of Mg^2+^ ions, and aqueous MgCl_2_ solutions were prepared over a broad range of molar concentrations.

DNA powder was dissolved in previously prepared MgCl_2_ solutions and incubated for 48 h at 4 °C with occasional stirring to ensure complete dissolution and formation of homogeneous solutions. In total, fifteen stock solutions were prepared by combining three DNA concentrations with five different magnesium-to-phosphate molar concentration ratios, defined as r = [Mg]/[P]: r = 0.1, 0.5, 1, 1.5 and 2.

To obtain thin films, 30 μL aliquots of the respective DNA stock solutions were deposited onto optical-grade silicon transmission windows and dried in a desiccator for 10 min under reduced pressure. Drying was performed using a Leybold S1 rotary vacuum pump equipped with a Thermovac TTR91N vacuum gauge (Leybold GmbH, Köln, Germany). The pressure inside the desiccator was recorded every 30 s, ensuring identical preparation conditions for all samples [15,16].

Drying under active vacuum provides significantly improved control over thin-film formation compared to conventional air-drying procedures commonly used in attenuated total reflectance (ATR) and other infrared techniques. This approach ensures reproducible preparation conditions—specifically pressure, temperature, and drying time—thereby minimizing spectral variability. In addition, the experimental setup, specifically optimized for DNA thin-film studies, reduces uncertainties associated with water subtraction, particularly in the base vibration region.

### 4.2. FTIR Measurements and Data Processing

FTIR spectra of DNA thin films were recorded using a PerkinElmer Spectrum GX spectrometer (Waltham, MA, USA) equipped with a nitrogen-cooled mercury–cadmium–telluride (MCT) detector and a KBr beam splitter. Measurements were performed in transmission mode at 25 °C with a spectral resolution of 4 cm^−1^ and 32 co-added scans.

Raw spectra were processed using the Kinetics add-on for MATLAB 2010 (MathWorks, Natick, MA, USA). Baseline correction and normalization were performed using the band at 966 cm^−1^ (deoxyribose C–C stretching mode) as an internal reference, as this band exhibited negligible changes in position and intensity across all samples. For each of the fifteen solution types, ten spectra were recorded and subsequently averaged using EssentialFTIR 3.5 software (Monona, WI, USA). Standard deviations were calculated using OriginPro 2019 (OriginLab Corp., Northampton, MA, USA). Repeated measurements showed point-by-point relative standard deviations typically below 10% of band intensity, well below the magnitude of the observed ion-induced spectral changes.

## Figures and Tables

**Table 1:** Assignments of major bands found in FTIR spectrum of ultra-short DNA thin film in the sugar-phosphate region from 1300–800 cm^−1^. Bands at approximately 1232, 1185, 893, 860, 837 and 805 cm^−1^ are considered conformation-related bands (see [15,16,18,19] and the references cited therein).

Band Position/cm^−1^	Assignment
1703	C=O
1686	C=O
1471	C=N
1232	Asymmetric PO_2_^−^
1185	C_3_′ endo-sugar phosphate
1089	Symmetric PO_2_^−^
1064	C-O phosphodiester
967	Deoxyribose C-C
893	Deoxyribose
860	C_3_′ endo-sugar, A-form
837	C_2_′ endo-sugar, B-form
805	C_3_′ endo-sugar, A-form

## Data Availability

All data supporting the findings of this study are available within this article. Additional raw data and analytical method validation details are available from the corresponding authors upon reasonable request.

## Abbreviations

ATR, attenuated total reflection; B-DNA, B-form DNA; A-DNA, A-form DNA; bp, base pairs; dsDNA, double-stranded DNA; FTIR, Fourier transform infrared spectroscopy; MCT, mercury–cadmium–telluride detector; Mg^2+^, magnesium ion; Na^+^, sodium ion; PO_2_^−^, phosphate group; r, magnesium-to-phosphate ratio.
